# Genetic variation underlying cognition and its relation with neurological outcomes and brain imaging

**DOI:** 10.18632/aging.101844

**Published:** 2019-03-04

**Authors:** Maria J. Knol, Alis Heshmatollah, Lotte G.M. Cremers, M. Kamran Ikram, André G. Uitterlinden, Cornelia M. van Duijn, Wiro J. Niessen, Meike W. Vernooij, M. Arfan Ikram, Hieab H.H. Adams

**Affiliations:** 1 Department of Epidemiology, Erasmus MC University Medical Center, Rotterdam, the Netherlands; 2 Department of Neurology, Erasmus MC University Medical Center, Rotterdam, the Netherlands; 3 Department of Radiology and Nuclear Medicine, Erasmus MC University Medical Center, Rotterdam, the Netherlands; 4 Department of Internal Medicine, Erasmus MC University Medical Center, Rotterdam, the Netherlands; 5 Department of Medical Informatics, Erasmus MC University Medical Center, Rotterdam, the Netherlands; 6 Faculty of Applied Sciences, Delft University of Technology, Delft, the Netherlands; * Equal contribution

**Keywords:** cognition, cognitive reserve, genetics, neuroimaging, neurological disorders

## Abstract

Cognition in adults shows variation due to developmental and degenerative components. A recent genome-wide association study identified genetic variants for general cognitive function in 148 independent loci. Here, we aimed to elucidate possible developmental and neurodegenerative pathways underlying these genetic variants by relating them to functional, clinical and neuroimaging outcomes. This study was conducted within the population-based Rotterdam Study (N=11,496, mean age 65.3±9.9 years, 58.0% female). We used lead variants for general cognitive function to construct a polygenic score (PGS), and additionally excluded developmental variants at multiple significance thresholds. A higher PGS was related to more years of education (β=0.29, p=4.3x10^-7^) and a larger intracranial volume (β=0.05, p=7.5x10^-4^). To a smaller extent, the PGS was associated with less cognitive decline (β_ΔG-factor_=0.03, p=1.3x10^-3^), which became non-significant after adjusting for education (p=1.6x10^-2^). No associations were found with daily functioning, dementia, parkinsonism, stroke or microstructural white matter integrity. Excluding developmental variants attenuated nearly all associations. In conclusion, this study suggests that the genetic variants identified for general cognitive function are acting mainly through the developmental pathway of cognition. Therefore, cognition, assessed cross-sectionally, seems to have limited value as a biomarker for neurodegeneration.

## Introduction

General cognitive function represents the ability to perform tasks across different cognitive domains. The development of the nervous system shapes an important part of the inter-individual variation in cognitive performance, with neurodegenerative processes increasingly contributing later in life [1,2]. As such, general cognition is a mixed construct consisting of both developmental and degenerative components [1], of which the neurodegenerative element may serve as an endophenotype for clinical outcomes such as daily functioning, dementia, parkinsonism, and stroke.

Recently, the highly polygenic architecture of general cognitive function was partly elucidated by the identification of 178 lead genetic variants in 148 independent loci [3]. However, it is unclear whether these variants act through a developmental or neurodegenerative pathway. Elucidating these pathways could provide more insight into the underlying biology of cognition and its potential as an endophenotype for clinically relevant outcomes. A developmental pathway would be more likely when these variants are linked to markers of cognitive and brain reserve such as educational attainment. On the other hand, more evidence for a neurodegenerative pathway would be gained when the variants are associated with clinical outcomes and brain imaging markers linked to neurodegeneration or accelerated cognitive decline.

Thus, in this population-based study, we aimed to elucidate the possible underlying pathways of the recently identified genetic variants for general cognitive function by exploring their associations with cognitive decline, measures of daily functioning, the risk of neurological disorders, and (micro)structural neuroimaging.

## RESULTS

**Figure 1:**
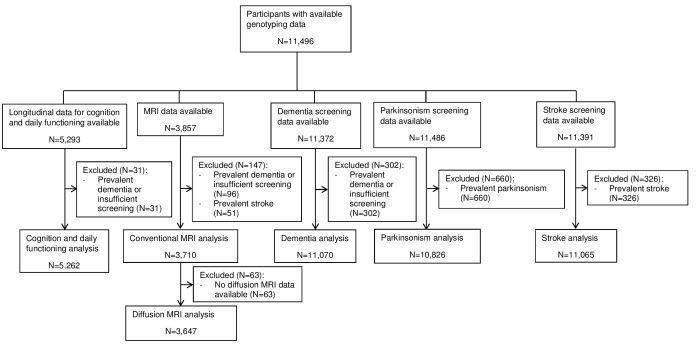
**Flowchart presenting the in- and exclusions of participants in the different analyses.** Abbreviations: magnetic resonance imaging (MRI).

Genotyping data was available for 11,496 individuals with a mean age of 65.3±9.9 years, of which 58.0% were women. A flowchart for the inclusion of participants in the different analyses is shown in Figure 1. Table 1 contains an overview of the study population characteristics for the different analyses.

**Table 1:** Study characteristics*.

**Characteristic**	**Sample**									
	**Cognition and ADL** **N_total=** **5,262**	**Brain imaging** **N_total=** **3,710**	**Dementia**		**Parkinson’s disease**		**Parkinsonism**		**Stroke**	
			**N_total=** **11,070**	**N_cases=** **1,444**	**N_total=** **10,588**	**N_cases=** **126**	**N_total=** **10,826**	**N_cases=** **258**	**N_total=** **11,391**	**N_cases=** **1,220**
**Age, years**	64.0±9.1	64.0 (11.0)	64.8±9.5	72.0±8.0	64.6±9.4	69.2±8.7	64.9±9.7	70.7±8.8	65.1±9.8	70.4±8.7
**Female, % (N)**	57.4 (3,022)	55.0 (2,039)	57.6 (6,376)	68.0 (982)	57.3 (6,065)	46.8 (59)	57.4 (6,219)	52.3 (135)	58.2 (6,436)	58.9 (718)
**Follow-up time, years**	6.1±0.6	-	12.2±6.4	11.3±6.3	12.4±6.5	7.8±5.9	12.3±6.5	7.7±5.8	12.3±6.6	9.4±5.9


*Values are expressed in mean±standard deviation unless stated otherwise; N_total is the total number of people for whom this characteristic is assessed; N_cases is the number of cases.

Abbreviations: activities of daily living (ADL).

### Cognitive performance and daily functioning

**Figure 2:**
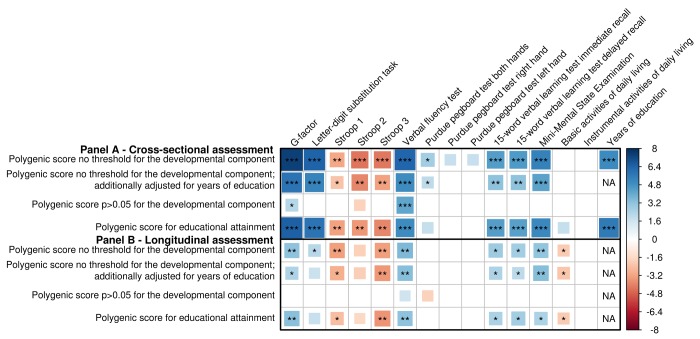
**Association of genetic variants for general cognitive function with (decline in) cognition and daily functioning, and educational attainment.** Association between genetic variants and cognitive performance and daily functioning at one point in time, as well as years of education, adjusted for age and sex with and without adjustment for years of education (**A**), and change in cognitive performance and daily functioning over time (**B**), additionally adjusted for baseline measurement and time between baseline and follow-up measurement. Three polygenic scores are presented: a cognition polygenic score including all independent lead variants (N=170); a cognition polygenic score only including variants with a p>0.05 for the association with the developmental component of cognition, i.e. educational attainment and intracranial volume (N=36); and an educational attainment polygenic score, which contains the lead genetic variants for cognitive performance (N=170) but uses the weights for educational attainment. Larger blocks indicate higher t-values. Higher scores indicate better performance, except for the Stroop test, the Basic Activities of Daily Living and Instrumental Activities of Daily Living. Significance levels are indicated by asterisks: *p<0.05, nominally significant; **p<0.0038 (**A**) or p<0.0040 (**B**), adjusted for the number of independent traits as calculated through 10,000 permutations; ***p<2.2x10^-5^ (**A**; 0.0038/170) or p<2.4x10^-5^ (**B**; 0.0040/170), additionally adjusted for the number of genetic variants.

As a methodological validation, we looked whether a cross-sectional relation was present between the polygenic score (PGS) and cognition. Indeed, an increase in the PGS was significantly associated with a higher general cognitive performance (‘G-factor’) (β=0.08, p=1.2x10^-13^), as well as with individual cognitive tests (Figure 2A). The PGS was also significantly associated with more years of education (β=0.29, p=4.3x10^-7^). No associations with daily functioning were found. Adjusting for years of education caused an attenuation of the associations, yet only the associations for Stroop 1 became non-significant. Nearly all associations attenuated after removing variants associated with the developmental component of cognition (Figure 2A, Supplementary Figure S1). To explore the developmental component further, we created a PGS of the same 170 genetic variants using the weights for educational attainment. This PGS showed similar associations with all cognitive tests. No individual variant was significantly associated with any of the outcomes. All results for the cross-sectional analyses of cognition, daily functioning and education are shown in Supplementary Table S3.

Figure 2B shows that a higher PGS was associated with less cognitive decline (β_ΔG-factor_=0.03, p=1.3x10^-3^), although this association became non-significant after adjusting for years of education (β_ΔG-factor_=0.02, p=1.6x10^-2^). A higher PGS was also associated with less decline in basic activities of daily living (BADL), although this was not significant after correcting for multiple comparisons (β=-0.02, p=4.4x10^-2^). Removing genetic variants associated with educational attainment and intracranial volume did not show a substantial enrichment of the effects. In contrast, the PGS for educational attainment even showed a slightly stronger association with cognitive decline than the cognition PGS (β_ΔG-factor_=0.03, p=8.3x10^-4^) (Figure 2B, Supplementary Figure S2). In the single-variant analysis, no variant reached statistical significance. MMSE measurements were available in a larger sample (N=9,369) with up to six measurements and a maximum follow-up of 25.2 years. In this sample, we observed a modest but significant relation between the PGS and yearly Mini-Mental State Examination (MMSE) change using linear mixed models (β=3.5x10^-3^, p=4.3x10^‑4^). Supplementary Table S4 contains the complete results for the longitudinal analyses for cognition and daily functioning.

### Clinical outcomes

**Figure 3:**
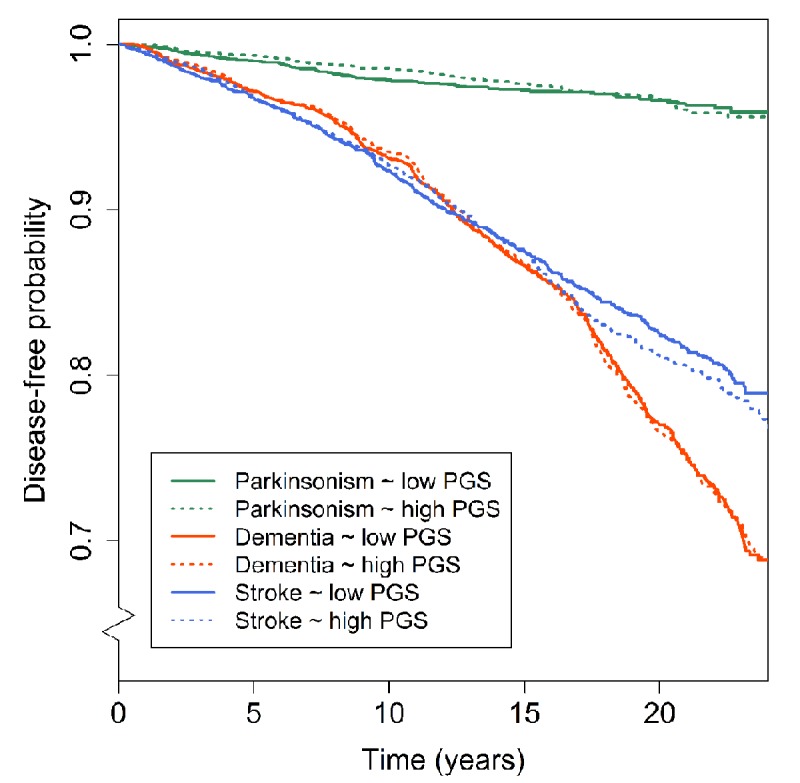
**Polygenic scores for general cognitive function and disease-free probability for dementia, parkinsonism and stroke.** Kaplan-Meier curves presenting the association between low (i.e. below the median) and high (above the median) polygenic scores and the disease-free probability over time for dementia, parkinsonism, and stroke. Abbreviations: polygenic score (PGS).

No significant association was found between the PGS and any of the clinical outcomes (Figure 3). Out of all 170 individual lead variants, none was significantly associated with the risk of one of dementia, parkinsonism or stroke. An increased risk for dementia was found after excluding variants associated with the developmental component at a p>0.05 threshold (hazard ratio 1.06, p=0.040), although this did not survive correction for multiple testing (Supplementary Figure S3). The PGS for educational attainment was not related to any of the neurological outcomes. Full results for the analyses of clinical outcomes are presented in Supplementary Table S5.

### Brain imaging markers

**Figure 4:**
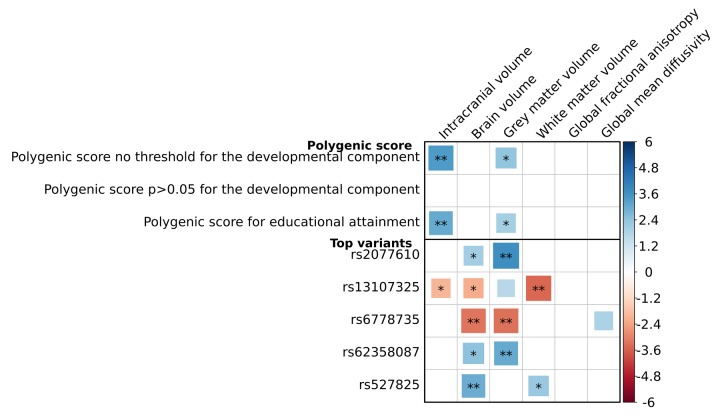
**Association between genetic variants for general cognitive function and global brain imaging markers.** Association between genetic variants for general cognitive function and both volumetric and global white matter microstructural integrity markers. For the volumetric outcomes, analyses were adjusted for age and sex, and additionally for intracranial volume if the outcome was not intracranial volume. For the microstructural integrity outcomes, analyses were adjusted for age, sex, white matter and white matter lesion volume. Three polygenic scores are presented: a cognition polygenic score including all independent lead variants (N=170); a cognition polygenic score only including variants with a p>0.05 for the association with the developmental component of cognition, i.e. educational attainment and intracranial volume (N=36); and an educational attainment polygenic score, which contains the lead genetic variants for cognitive performance (N=170) but uses the weights for educational attainment. Also, the five top genetic variants for the association with these brain imaging markers are presented. Positive associations depicted in blue correspond to a larger volume or a better white matter microstructural integrity. Larger blocks indicate higher t-values. Significance levels are indicated by asterisks: *p<0.05, nominally significant; **p<0.0101, adjusted for the number of independent traits as calculated through 10,000 permutations. No association was significant after additional adjustments for the number of genetic variants tested (p<5.9x10^-5^; 0.0101/170).

**Figure 5:**
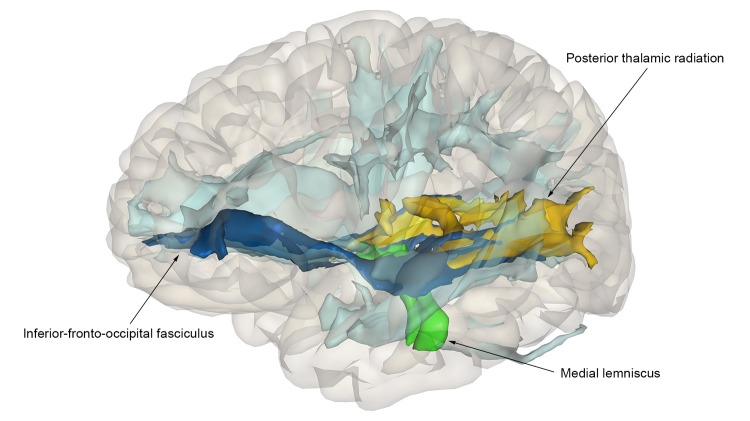
**Association of polygenic scores for cognition and tract-specific diffusion-MRI measures.** Nominally significant tracts are color-coded: dark-blue – inferior-fronto-occipital fasciculus; green – medial lemniscus; yellow – posterior thalamic radiation. Non-significant tracts are colored in light-blue.

We found that a higher PGS was significantly related to a larger intracranial volume (β=0.05, p=7.5x10^-4^), but not with the other volumetric measures or with global white matter microstructural integrity (Figure 4). At a nominal significance level, a higher PGS was associated with a higher fractional anisotropy (FA) in the medial lemniscus, and a lower mean diffusivity (MD) in the inferior-fronto-occipital fasciculus and the posterior thalamic radiation (minimal p=2.2x10^-2^), but this did not survive correction for multiple testing (Figure 5). Removing genetic variants associated with the developmental component of cognition did not show a pattern of enrichment of the associations. The associations between the educational attainment PGS with the brain imaging markers were comparable to those of the cognition PGS (Figure 4, Supplementary Figure S4-S5). No individual variant reached the significance threshold for the association with any of the brain imaging markers after multiple comparisons correction. Full results of the brain imaging analyses can be found in Supplementary Table S6, Supplementary Table S7

## DISCUSSION

In this population-based study among middle-aged and elderly persons, a PGS based on recently identified genetic variants for global cognition was associated with better global and test-specific cognitive performance, more years of education and a larger intracranial volume. This PGS was also associated with measures of cognitive decline, although these associations attenuated after adjusting for educational attainment, and no enrichment of the effects was observed when we excluded variants associated with developmental cognitive components. We did not find significant associations with (decline in) daily functioning, the incidence of dementia, parkinsonism or stroke, or with other brain imaging markers.

Cognitive decline is considered an important marker for the development and progression of neurodegenerative diseases [4–6]. However, we found that a higher cognition PGS was mainly associated with a better cognitive performance cross-sectionally, and only to a limited extent longitudinally. In fact, a PGS of the same variants using the weights for educational attainment was equally or more associated with cognitive decline. In contrast, we did find associations with developmental components of cognition, i.e. educational attainment and intracranial volume. Brain and cognitive reserve are partially determined by genetics and are suggested to be protective against cognitive decline [7–10]. It is also seen as an explanation for interindividual differences in the clinical presentation of neurodegenerative diseases in patients with a similar neuropathology [11–13]. These findings are thus suggestive of a developmental pathway underlying the genetic variants for cognitive performance.

To our knowledge there are as yet no other studies that have investigated the association between these genetic variants and clinical outcomes. We found no significant relation between the PGS and the risk of dementia, parkinsonism or stroke. If anything, we observed a nominally significant association with the incidence of dementia after we excluded all genetic variants associated with the developmental cognitive component (p<0.05). However, the direction of effect was not as expected, i.e. a higher PGS – associated with better cognitive function – showed an increase in the risk of dementia. Yet, since this association did not survive correction for multiple testing, no strong conclusions should be drawn from this finding, and validation in other studies is needed. Previous observational studies have shown associations between cognitive function and dementia, parkinsonism, Parkinson’s disease and stroke, both before and after diagnosis [5,14–18]. Also, disease-specific genetic variants for these disorders have been associated with cognitive functioning [19–22]. This may indicate that cognitive decline as seen in abovementioned (prodromal) clinical outcomes is mainly caused by disease-specific variants rather than variants for general cognitive function. However, we also did not find significant associations between the cognition PGS and diffusion magnetic resonance imaging (MRI) measures, in contrast to previous studies that showed associations between global and tract-specific microstructural integrity and cognition and cognitive decline [23–26]. This may indicate that these associations are mainly driven by non-genetic components. Another possibility is that our study did not have enough power to detect associations with the incidence of the clinical outcomes and neuroimaging measures. Alternatively, there may be effects of the genetic variants not seen on traditional structural neuroimaging; future studies on other imaging markers such as functional MRI could therefore prove useful information. Due to the absence of an association between the PGS and clinical outcomes and the modest association with cognitive decline, we attempted to enrich the degenerative component of the PGS by filtering out genetic variants that are associated with intracranial volume and educational attainment. By applying this filter, nearly all associations for the different analyses attenuated, supporting the suggestion that the genetic variants mainly represent the developmental component of cognitive performance. However, removing genetic variants associated with the developmental component may also eliminate degenerative components of the PGS if some variants are pleiotropic, possibly leading to an underpowered study for detecting an effect of the PGS. A more robust method would be to perform a genome-wide association study (GWAS) with cognitive decline as an outcome instead of using cross-sectional measurements of cognitive performance, preferably in an elderly population since neurodegeneration mainly occurs later in life. However, longitudinal measurements such as those in the present study are only available in a fraction of the samples with cross-sectional assessments, which at present impedes GWAS discoveries for cognitive decline.

Strengths of this study are the population-based setting, the longitudinal assessment of cognitive function and daily functioning, the availability of structural brain imaging, and the long follow-up period for dementia, parkinsonism and stroke. We also need to consider limitations. It should be noted that the Rotterdam Study was part of the discovery sample for the general cognitive function, educational attainment and intracranial volume GWAS [3,9,27]. However, for cognitive function and educational attainment, this was only a small proportion of the total sample size (2.0% and 1.4%, respectively), yet for ICV this was a larger percentage (18.2%). However, we only included genome-wide significant variants and these will most likely not be different if the Rotterdam Study would be excluded from the meta-analysis. Moreover, most variants were excluded due to their association with educational attainment (94.0%), thus we do not expect that this influenced our findings to a large extent. Another limitation is that the effect estimates in the summary statistics of the GWAS are based on the effect estimates of many different populations, and they may not be the correct estimates for the Dutch population as present in the Rotterdam Study. In addition, the self-reported years of education may not be the best measure of educational attainment since the type and level of education is not taken into account. Also, cognitively impaired participants may not correctly recall their received education, possibly creating noise in this variable. Furthermore, the mean follow-up time of 6.1 years for the cognitive tests in this study is relatively short, which limits the power of detecting associations with cognitive decline. Additionally we assumed a linear decay of cognition over time. Despite this, we observed similar associations with cognitive decline when studying the association between the PGS change in MMSE using linear mixed models in a sample with up to six measurements. Selection bias may have occurred since cognitively impaired participants are less likely to visit the examination center, which may have caused an underestimation of the true association with cognitive decline. However, for the clinical outcomes, this selection bias is less likely to have occurred since the study database was linked to the participants’ medical records.

In conclusion, we found that a PGS for general cognitive function was associated with cognitive performance, intracranial volume and educational attainment, and to a limited extent with cognitive decline. We found no associations between the PGS and daily functioning, neurological disorders, or global brain tissue volumes and diffusion-MRI measurements. Using the weights of the educational attainment GWAS, similar associations were observed. Removing variants associated with developmental components of cognition did not cause a substantial enrichment of the associations with neurodegenerative outcomes. Based on our results we postulate that the genetic variants identified for general cognitive function are acting mainly through the developmental pathway of cognition. Therefore, cognition, assessed cross-sectionally, seems to have limited value as a biomarker for neurodegeneration. Future studies that focus on identifying genetic variants specific for cognitive decline are needed to help understand the pathophysiology underlying the degenerative component of cognition.

## MATERIALS AND METHODS

### Study population

This study was conducted within the Rotterdam Study, an ongoing population-based cohort study in the Netherlands with the aim to investigate causes and determinants of diseases in the elderly [28]. This cohort was initiated in 1990 and extended in 2000 and 2006, with a total of 14,926 participants aged 45 years and older who undergo examinations every three to four years. Assessment of dementia, parkinsonism and stroke has been performed since the start of the study. In 2002, an extensive cognitive test battery was added to the core protocol. MRI scanning was implemented in the study protocol from 2005 onwards [29]. Out of 14,926 subjects, genotyping was successfully performed in 11,496 participants. Figure 1 gives an overview of the selection of participants for the different analyses, presented in a flowchart. According to the Population Study Act Rotterdam Study, the Ministry of Health, Welfare and Sports of the Netherlands has given approval for the Rotterdam Study. All participants have given written informed consent [28].

### Outcome selection

Outcomes were selected based on their link with either development or neurodegeneration. Educational attainment and intracranial volume (ICV) are established markers of cognitive and brain reserve and can therefore be used to study the developmental component of cognition [30,31]. On the other hand, dementia, parkinsonism, and stroke are clinical outcomes related to accelerated cognitive decline and neurodegeneration [5,14–16]. Furthermore, daily functioning, global brain tissue volumes, and diffusion-MRI measurements have been associated with impaired cognition in the elderly [4,16,23], and can be used as a marker for neurodegeneration.

### Genotyping

The Illumina 550K, 550K duo and 610 quad arrays were used for genotyping. Samples with a call rate below 97.5% were removed, as well as gender mismatches, excess autosomal heterozygosity, duplicates or family relations, ethnic outliers, variants with call rates lower than 95.0%, failing missingness test, Hardy-Weinberg equilibrium p-value smaller than 10^-6^ and allele frequencies smaller than 1%. Genotypes were imputed using MaCH/minimac software to the 1000 Genomes phase I version 3 reference panel.

### Polygenic scores

We calculated a PGS using the lead genetic variants with their corresponding effect sizes for general cognitive function [3]. Genetic variants that were not available in the reference panel and variants with an r^2^<0.30 were excluded (N=7 and N=1, respectively). For the remaining genetic variants (N=170), the allele dosage was multiplied by the reported effect estimate (Supplementary Table S1). Subsequently, the weighted effects of all variants were added up and the resulting PGSs were standardized into Z-scores.

Since we aimed to differentiate the developmental component of general cognitive function from degenerative effects, we calculated additional PGSs where variants associated with educational attainment and intracranial volume were removed at multiple p-value thresholds. For each variant, we used the lowest p-value threshold for either educational attainment or intracranial volume. The p-values were extracted from the summary statistics of a GWAS on educational attainment performed in a discovery sample of 766,345 individuals [27], and a GWAS on intracranial volume performed in a discovery sample of 26,577 individuals [32]. The different p-value thresholds for the association with educational attainment and intracranial volume, with the corresponding number of variants that remained, as well as the explained variance of the G-factor in our dataset are shown in Supplementary Table S2. When applying the strictest p-value threshold for the exclusion of developmental variants (p>0.05), 36 genetic variants remained.

Additionally, to explore the developmental component of the lead genetic variants for general cognitive function further, we created PGSs of the same 170 variants using the weights of the educational attainment GWAS (Supplementary Table S1).

### Cognitive test battery

For the cognition and daily functioning analyses, only participants who had two measurements for at least one of the tests underlying these outcomes were included. The MMSE was assessed as a measure of global cognitive function, and was collected since the initiation of the Rotterdam Study. From 2002 onwards, cognitive function was additionally assessed using multiple cognitive tests: the 15-word verbal learning test (15-WLT), the Stroop test (consisting of reading, color naming and interference tasks, error-adjusted scores), the letter-digit substitution task (LDST), the verbal fluency test (using animal categories) and the Purdue pegboard (PPB) test for the left hand, right hand and both hands [2,33–36]. A measure of general cognitive function (‘G-factor’) was obtained through principal component analysis on the delayed recall score of the 15-WLT, Stroop interference test, LDST, verbal fluency task and the PPB test, as described previously [2]. The G-factor explained 53.4% and 51.9% of the variance in cognitive test scores in our population at baseline and follow-up visit, respectively. Z-scores were calculated in order to make comparable test results.

Self-reported years of education was used as a measure of educational attainment.

### Assessment of daily functioning

Two components of daily functioning were assessed: BADL and instrumental activities of daily living (IADL). The Dutch version of the Stanford Health Assessment Questionnaire was used to measure BADL [37], and IADL was measured using the Dutch version of the IADL scale [38]. To prevent selective loss of data, IADL items scored as non-applicable were imputed using the variables age, sex, BADL scores and all other available IADL items. Both BADL and IADL scores were standardized into Z-scores.

### Assessment of clinical outcomes

The assessment of dementia, parkinsonism (including Parkinson’s disease) and stroke have previously been described in detail [39–41]. In summary, history of these clinical outcomes was assessed during the baseline interview. Participants were screened at baseline and subsequent center visits for dementia with the MMSE and the Geriatric Mental Schedule organic level, and for signs of parkinsonism. Participants with a positive screening were further examined and were evaluated by a panel led by an experienced neurologist who made the definitive diagnosis. After enrollment, participants were continuously monitored for dementia, parkinsonism and stroke through automated linkage of the study database with files from general practitioners. Follow-up for parkinsonism (including Parkinson’s disease) was available until the 1^st^ January 2015 and for dementia and stroke until the 1^st^ January 2016.

### MRI acquisition and processing

We performed a multi-sequence brain MRI scan on a 1.5 tesla research dedicated MRI scanner (GE Signa Excite). Imaging details are provided elsewhere [29]. In short, the scan protocol included a T1-weighted image, a T2-weighted fluid-attenuated inversion recovery (FLAIR) sequence, a proton density weighted image and a spin echo echo planar diffusion weighted image for the diffusion-MRI. A multimodal algorithm was used based on T1-weighted, T2-weighted and FLAIR images to segment voxels into grey matter, white matter, white matter lesion volume, cerebrospinal fluid and background tissue using a k-nearest-neighbor-algorithm trained on six manually labelled atlases [42,43]. We estimated supratentorial intracranial volume by summing total grey and white matter volume and cerebrospinal fluid [42].

For the diffusion-MRI, three volumes were performed without diffusion weighting of which the average was used (b-value=0 s/mm^2^, maximum b-value was 1000 s/mm^2^). Diffusion tensors were computed using ExploreDTI to obtain FA and MD in normal-appearing white matter voxels. We segmented fifteen white matter tracts using probabilistic tractography and atlas-based masking [44]. Tracts were grouped based on anatomic location or presumed function into brain stem tracts, projection tracts, association tracts, limbic system tracts and callosal tracts. Tract-specific FA and MD but also white matter volumes and white matter lesion volumes in specific tracts were obtained as previously described [44]. In general, a lower FA and a higher MD are indicative of lower microstructural white matter integrity.

### Data analysis

Linear regression models were used to assess the associations between the PGS and cognition, daily functioning, volumetric brain outcomes and white matter microstructural integrity. For the cross-sectional analysis of cognition and daily functioning we analyzed the first measurement. We additionally assessed the association with change in MMSE using linear mixed models with an interaction between the PGS and time. We used a random intercept and slope for time and included participants with a minimum of two MMSE measurements. Cox proportional hazard models were used to study the association between the PGS and the incidence of dementia, parkinsonism, and stroke. The proportional hazards and linearity assumptions were met. All models were adjusted for age and sex. Models assessing cognition and daily functioning were performed with and without adjustment for educational attainment. Longitudinal cognition and daily functional analyses were adjusted for time between baseline and follow-up visit, and additionally for baseline measurements in the linear regression analyses. Volumetric brain outcomes were adjusted for intracranial volume when the outcome was not intracranial volume, and additionally for white matter and white matter lesion volume in the analyses for white matter microstructural integrity. The abovementioned analyses were repeated for all genetic variants separately.

Since outcomes for the different analyses may be correlated, we used permutation testing in order to assess the number of independent outcomes for each subsection. Based on this information, we defined the multiple testing p-value thresholds for the different analyses, namely p<0.0038 for the cross-sectional and p<0.0040 for the longitudinal analyses of cognitive performance and daily functioning; p<0.0101 for the volumetric and global diffusion-MRI brain measures, and p<0.0022 for the tract-specific diffusion-MRI analyses; and p<0.0129 for the clinical outcomes. For the analyses of the genetic variants separately, we additionally used the Bonferroni correction for multiple testing, using the formula k/170 with k representing the p-value threshold as obtained by permutation testing. Analyses were performed using the IBM SPSS Statistics 21 and R 3.4.0 software.

## Supplementary Material

Supplementary Figures

Supplementary Table S1

Supplementary Table S2

Supplementary Table S3

Supplementary Table S4

Supplementary Table S5

Supplementary Table S6

Supplementary Table S7
