# Supplementary material for: Genetic variation underlying cognition and its relation with neurological outcomes and brain imaging
Source: Aging (Albany NY). 2019 Mar 4;11(5):1440–56. doi: 10.18632/aging.101844 (PMC6428100; doi:10.18632/aging.101844)
Supplement: Supplementary Figures [file aging-11-101844-s001.pdf]

SUPPLEMENTARY FIGURES

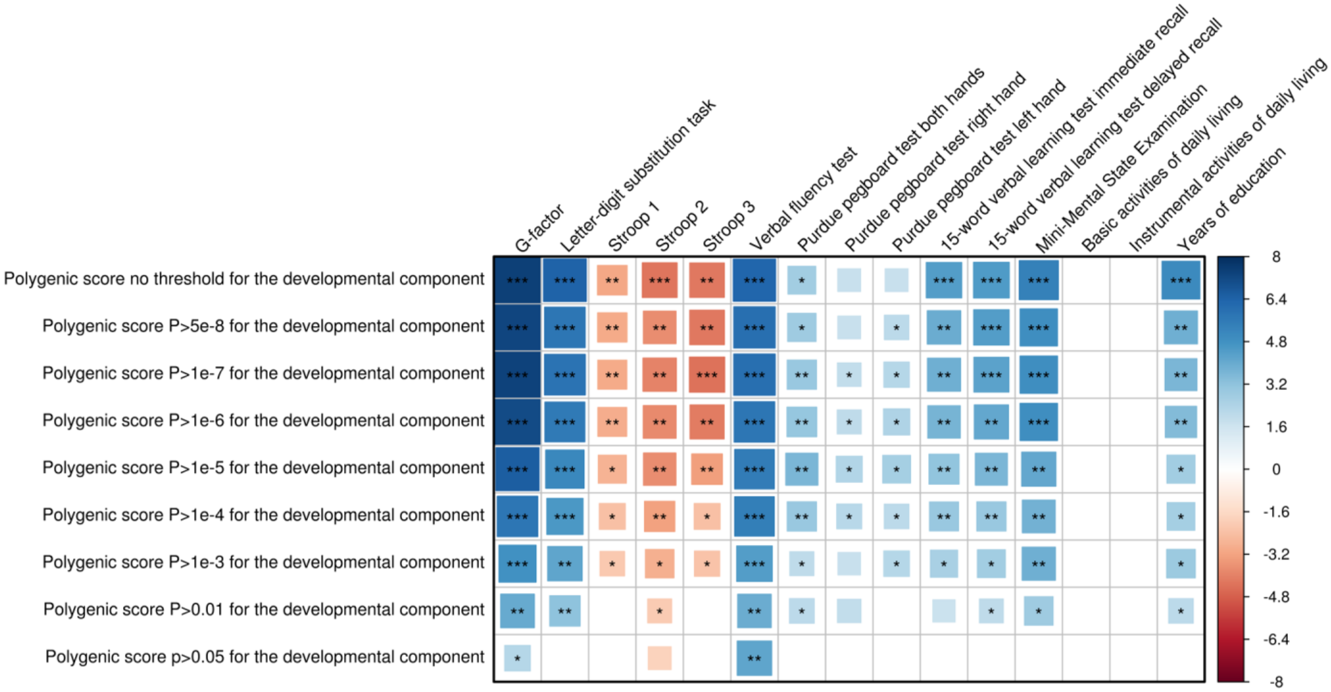

Association between polygenic scores for cognitive function and cognitive performance and daily functioning at one point in time, as well as years of education, adjusted for age and sex. Polygenic scores with different p-value thresholds for the exclusion of the developmental component are presented. For each variant we used the lowest p-value for either educational attainment or intracranial volume. Larger blocks indicate higher t-values. Higher scores indicate better performance, except for the Stroop test, the basic activities of daily living and instrumental activities of daily living. Significance levels are indicated by asterisks: *p<0.05, nominally significant; ***p<0.0038, adjusted for the number of independent traits as calculated through 10,000 permutations; ***p<2.2x10^-5 (0.0038/170), additionally adjusted for the number of genetic variants.

Supplementary Figure S1. Association between polygenic scores for general cognitive performance and cognition, daily functioning, educational attainment.

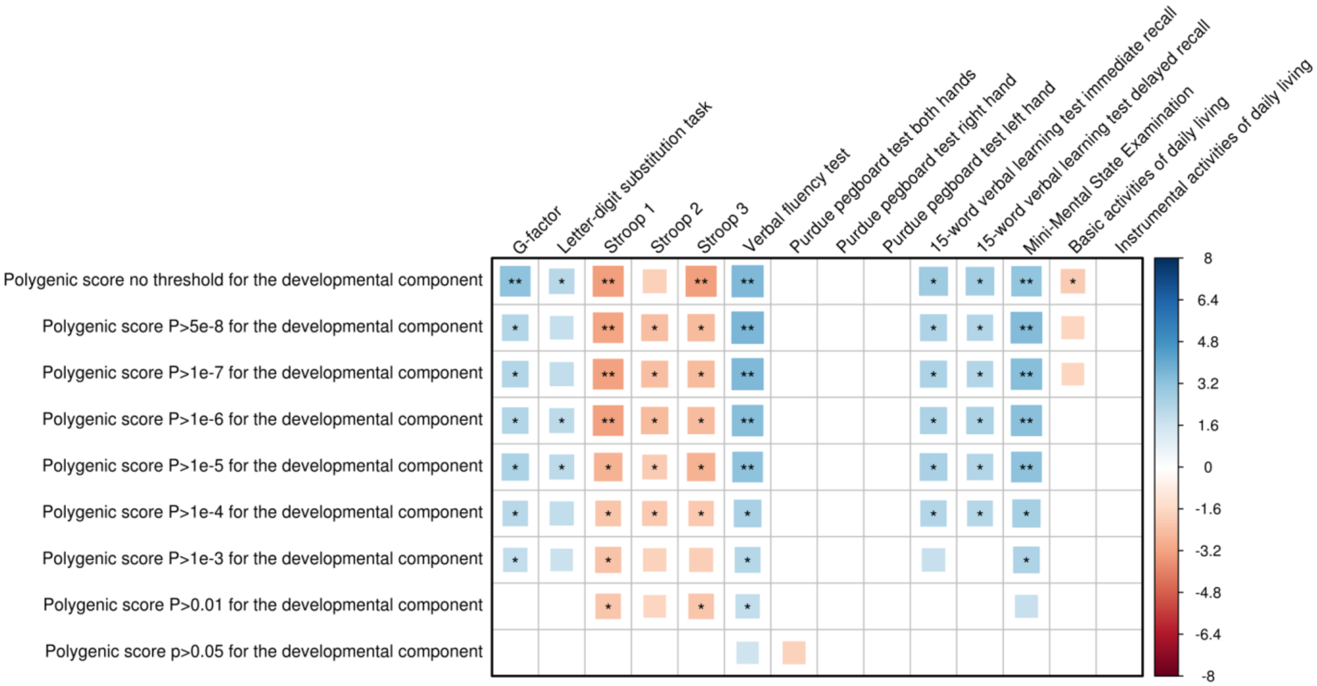

Association between polygenic scores for cognitive function and change in cognitive performance and daily functioning, adjusted for age, sex, baseline measurement and time between baseline and follow-up measurement. Polygenic scores with different p-value thresholds for the exclusion of the developmental component are presented. For each variant we used the lowest p-value for either educational attainment or intracranial volume. Larger blocks indicate higher t-values. Higher scores indicate better performance, except for the Stroop test, the basic activities of daily living and instrumental activities of daily living. Significance levels are indicated by asterisks: *p<0.05, nominally significant; **p<0.0040, adjusted for the number of independent traits as calculated through 10000 permutations.

Supplementary Figure S2. Association between polygenic scores for general cognitive performance and change in cognition and daily functioning.

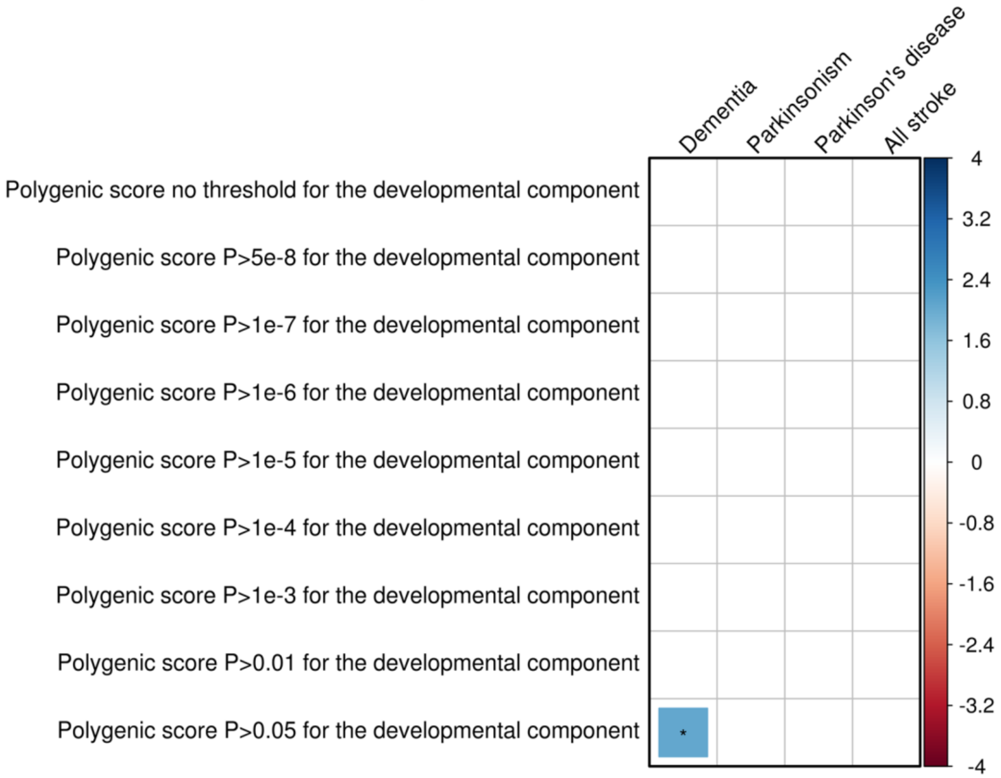

Association between polygenic scores for cognitive function and the incidence of dementia, parkinsonism, Parkinson’s disease and stroke assessed by Cox proportional hazards models, adjusted for age and sex. Polygenic scores with different p-value thresholds for the exclusion of the developmental component are presented. For each variant we used the lowest p-value for either educational attainment or intracranial volume. Larger blocks indicate higher Z-values. Significance levels are indicated by asterisks: *p<0.05, nominally significant. No association was significant after additional adjustments for the tested number of independent traits as calculated through 10,000 permutations (p<0.0129).

Supplementary Figure S3. Association between polygenic scores for general cognitive performance and the risk of neurologic disorders.

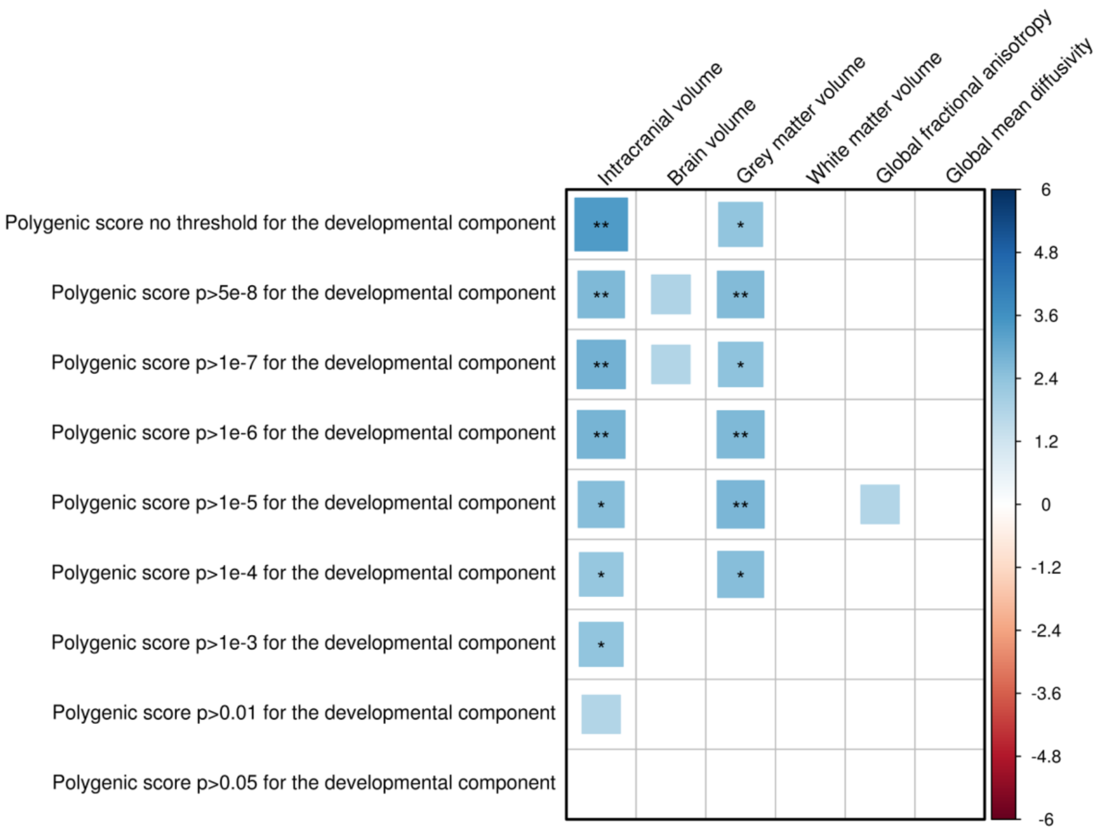

Association between polygenic scores for general cognitive function and several brain imaging markers. Polygenic scores with different p-value thresholds for the exclusion of the developmental component are presented. For each variant we used the lowest p-value for either educational attainment or intracranial volume. Positive associations depicted in blue correspond to a larger volume or a better white matter microstructural integrity. Larger blocks indicate higher t-values. Significance levels are indicated by asterisks: *p<0.05, nominally significant; **p<0.0101, adjusted for the number of independent traits as calculated through 10,000 permutations.

Supplementary Figure S4. Association between polygenic scores for general cognitive performance and volumentric ang gloal microstructural integrity brain imaging measures.

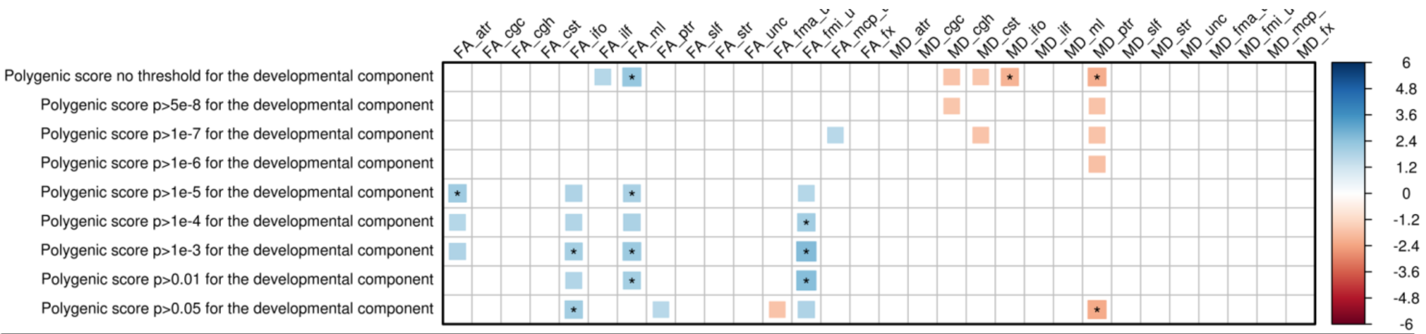

Association between polygenic scores for general cognitive function and tract-specific microstructural integrity. Polygenic scores with different p-value thresholds for the exclusion of the developmental component are presented. For each variant we used the lowest p-value for either educational attainment or intracranial volume. Positive associations depicted in blue correspond to a better white matter microstructural integrity. Larger blocks indicate higher t-values. Significance levels are indicated by asterisks: *p<0.05, nominally significant. No associations were significant after adjustment for the tested number of independent traits as calculated through 10,000 permutations (p<0.0022).
Abbreviations: anterior thalamic radiation (atr); cingulate gyrus part of cingulum (cgc); parahippocampal part of cingulum (cgh); corticospinal tract (cst); fractional anisotropy (FA); forceps major (fma_u); inferior fronto-occipital fasciculus (ifo); forceps minor (fmi_u); fornix (fx); inferior longitudinal fasciculus (ilf); medial cerebellar peduncle (mcp_u); mean diffusivity (MD); medial lemniscus (ml); posterior thalamic radiation (ptr); superior longitudinal fasciculus (slf); superior thalamic radiation (str); uncinated fasciculus (unc).

Supplementary Figure S5. Association between polygenic scores for general cognitive performance and tract-specific microstructural integrity.
